# Analysis of Safety from a Human Clinical Trial with Pterostilbene

**DOI:** 10.1155/2013/463595

**Published:** 2013-02-04

**Authors:** Daniel M. Riche, Corey L. McEwen, Krista D. Riche, Justin J. Sherman, Marion R. Wofford, David Deschamp, Michael Griswold

**Affiliations:** ^1^Department of Pharmacy Practice, The University of Mississippi School of Pharmacy, 2500 North State Street, Jackson, MS 39216, USA; ^2^Department of Medicine, The University of Mississippi Medical Center, 2500 North State Street, Jackson, MS 39216, USA; ^3^Department of Pharmacy, Cleveland Clinic, 9500 Euclid Avenue, Cleveland, OH 44195, USA; ^4^Department of Pharmacy, St. Dominic Hospital, 969 Lakeland Drive, Jackson, MS 39216, USA

## Abstract

*Objectives*. The purpose of this trial was to evaluate the safety of long-term pterostilbene administration in humans. *Methodology*. The trial was a prospective, randomized, double-blind placebo-controlled intervention trial enrolling patients with hypercholesterolemia (defined as a baseline total cholesterol ≥200 mg/dL and/or baseline low-density lipoprotein cholesterol ≥100 mg/dL). Eighty subjects were divided equally into one of four groups: (1) pterostilbene 125 mg twice daily, (2) pterostilbene 50 mg twice daily, (3) pterostilbene 50 mg + grape extract (GE) 100 mg twice daily, and (4) matching placebo twice daily for 6–8 weeks. Safety markers included biochemical and subjective measures. Linear mixed models were used to estimate primary safety measure treatment effects. *Results*. The majority of patients completed the trial (91.3%). The average age was 54 years. The majority of patients were females (71%) and Caucasians (70%). There were no adverse drug reactions (ADRs) on hepatic, renal, or glucose markers based on biochemical analysis. There were no statistically significant self-reported or major ADRs. *Conclusion*. Pterostilbene is generally safe for use in humans up to 250 mg/day.

## 1. Introduction

Pterostilbene is a phenol that is chemically related to resveratrol, a possible contributor to the “French Paradox” which associates red wine consumption and lower coronary heart disease [1, 2]. Naturally found in blueberries and grapes, pterostilbene is a phytoalexin, a class of compounds naturally synthesized by plants during pathogen infection. The primary structural difference between pterostilbene and resveratrol is that pterostilbene contains two methoxy groups and one hydroxyl group while resveratrol has three hydroxyl groups. The two methoxy groups cause pterostilbene to be more lipophilic, which increases oral absorption and gives pterostilbene a higher potential for cellular uptake [3]. Pterostilbene has a longer half-life (105 minutes versus 14 minutes) and higher oral bioavailability (80% versus 20%) compared to resveratrol [4–7]. Pterostilbene also has low total body clearance and subsequent Vss which suggests extensive tissue distribution [4].

There has been extensive animal research examining both the safety and efficacy of pterostilbene. Animal studies have demonstrated efficacy in cardiometabolics (e.g., cholesterol and blood glucose), as well as cancer and cognition mediators [8–10]. 

Substances that are generally recognized as safe (GRAS) are exempt from premarket Food and Drug Administration (FDA) review and may be intentionally added to food. The criteria for GRAS status are described in sections 201(s) and 409 of the Food Additives Amendment to the Federal Food, Drug, and Cosmetic Act in 1958 [11]. After 1958, any food substance must be scientifically evaluated by experts and deemed safe for human consumption to attain the GRAS recognition [11].

A short-term, open-label trial conducted in 13 healthy volunteers evaluated the *in vivo* activity of a pterostilbene-rich extract (*Pterocarpus marsupium*). Safety parameters were evaluated through blood drawn. No changes from baseline parameters were demonstrated. There were also no observed adverse events of major body systems [12]. This healthy volunteer trial did not examine synthesized or pure pterostilbene with a blinded control and did not investigate long-term safety in a target patient population.

Our trial is the first controlled trial performed in humans evaluating the safety of pterostilbene. 

## 2. Methodology

This trial was a prospective, randomized, double-blind placebo-controlled intervention trial. This trial was approved by the University of Mississippi Medical Center Institutional Review Board. The target population was patients with hypercholesterolemia, defined as a baseline total cholesterol ≥200 mg/dL and/or, baseline low-density lipoprotein cholesterol ≥100 mg/dL. Patients were included if they were ≥18 years of age and on either no cholesterol therapy or cholesterol medication at a stable dose for at least 2 months prior to baseline laboratory. Patients were excluded if they had significant hepatic, renal, or gastrointestinal tract disease, were receiving thiazolidinediones or fibric acid derivatives, had current overt cardiovascular disease, were women of reproductive potential not receiving birth control, or were pregnant/nursing women. 

The trial planned for an enrollment of 80. Subjects were divided equally into one of four groups: pterostilbene 50 mg twice daily (low dose), pterostilbene 125 mg twice daily (high dose), pterostilbene 50 mg/grape extract 100 mg twice daily (low dose + grape extract), or matching placebo twice daily for 6–8 weeks. Ciprofibrate is a peroxisome proliferator-activated receptor-*α* (PPAR-*α*) agonist [8]. Pterostilbene has demonstrated similar PPAR-*α* activation at approximately equimolar concentrations in animal models [4]. Since a standard human dose of ciprofibrate is 100 mg/day, this dose was selected for the lowest effective pterostilbene dose in monotherapy and combination. The higher daily dose was evaluated to assess for potential dose-related efficacy or adverse effect. All patients received identical counseling on lifestyle intervention and compliance with currently prescribed medication regimens.

The manufacturer of the pterostilbene and placebo products was deemed in compliance by the FDA current good manufacturing practices prior to the initiation of this trial. The process of pterostilbene synthesis for this trial is described elsewhere [13].

The safety markers included biochemical and subjective measures collected at two visits (baseline and final). Donated blood was analyzed for all biochemical measures at the same laboratory values. Primary safety measures included Alanine Aminotransferase (ALT), Aspartate Aminotransferase (AST), serum creatinine, and blood glucose. Other safety markers include blood electrolytes and symptomatic subjective adverse drug reactions (ADRs) collected during patient interviews at baseline and final visits. Blood pressure, cholesterol, and weight were collected and reported separately as efficacy endpoints. Pill counts were utilized to assess for compliance. 

Linear mixed models were used to estimate primary safety measure treatment effects in order to account for intra subject associations arising from the repeated measures before and after longitudinal design. The underlying missing-at-random architecture implicit in mixed models was assumed. Various models were fit to examine potential baseline effects including as appropriate the following:

1. 3-way interaction models of final outcome × treatment group × baseline value;
2. 2-way interaction models including all 2-way terms from (1) but excluding the 3-way term;
3. models assuming baseline value affected change similarly across treatment groups;
4. models assuming change in outcome were independent of baseline value.

Each model was examined in unadjusted and adjusted form (adjusting for age, gender and race). Final reported treatment effects were obtained from the simplest appropriate adjusted model for each outcome. For secondary measures compared to baseline and/or placebo, a *t*-test was performed for continuous data and a Fisher's exact test was performed for dichotomous data.

## 3. Results

Patient demographics are detailed in Table 1. Over 90% of patients who enrolled completed the trial with an average duration of 52 days. Over 80% of trial completers demonstrated 80% or higher compliance based on pill counts. The average age of all trial participants was 54 years. The majority of patients were females and Caucasians. Among the pterostilbene groups (*n* = 60), 2 patients were lost to follow up and 2 patients withdrew from the trial. One withdrawal was due to a lost trial medication bottle and the other withdrawal was due to worsening of cholesterol from an outside laboratory.

**Figure 1:**
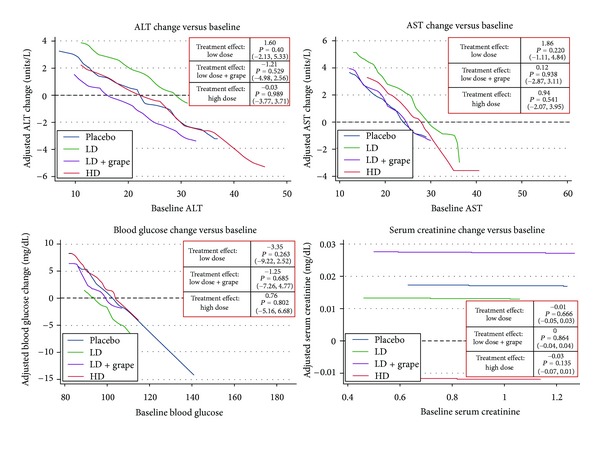
Primary safety analysis.* Interpretation:* expected Changes in an Outcome (vertical axis) for any given level of baseline value (horizontal axis) across all four treatment groups. Adjusted for age, gender, and race.

There was no biochemical ADRs on liver, kidney, or glucose markers (See Figure 1). There were no statistically significant self-reported ADRs versus placebo (see Table 2). There were no major ADRs (e.g., hospitalization, new-onset disease, infection, or death). There was a significant 3.6% reduction in bicarbonate in the high-dose group versus placebo (*P* = 0.02) with a similar trend in both low-dose groups. The combination of grape extract and low-dose pterostilbene decreased BUN by 7.1% from baseline (*P* = 0.01), but this reduction was not significant when compared to placebo (*P* = 0.20). There were no other significant effects on electrolyte markers.

Additionally, we performed a single-blinded quality assessment of 2 samples from 3 randomly selected bottles in each trial arm. The average amount of pterostilbene was 95% or higher of the listed active ingredient amount in all samples evaluated.

## 4. Discussion

This is the first well-designed comparison of pterostilbene in a dose-ranging controlled human trial. There appears to be no direct effect of pterostilbene on measures of hepatic or renal function. The proposed mechanism of action of pterostilbene is PPAR-*α* agonism [4]. Currently available FDA-approved PPAR-*α* agonists (e.g., fenofibrate or pioglitazone) have both renal and hepatic dose adjustments required. Fenofibrate has reported increases in serum creatinine from baseline by 12% as an ADR [14]. Despite a high prevalence of a combination with statin, pterostilbene did not demonstrate any biochemical hepatic ADRs. There does not appear to be a need for such precautions with pterostilbene in doses up to 250 mg/day.

No patients taking statins reported myopathy. Myopathy was reported in patients not taking statins in both low-dose groups on 3 occasions. The lack of myopathy in the high-dose group and in statin users decreases the likelihood of this ADR in relation to pterostilbene. Though a drug-drug interaction with statins appears unlikely, possible drug-drug interactions with other medication classes warrant further investigation.

There is unlikely an association of pterostilbene with gastrointestinal ADR (with or without food) or itching as both reported ADRs occurred to a low extent in only the placebo and high dose groups.

While <20% of completers reported any dietary changes during the trial, increased appetite was reported in all three pterostilbene arms, but not placebo. Although detailed changes in weight will be reported separately, there was no overall trend towards an increase in body weight. Participants reporting this ADR (*n* = 4) all gained weight (average 1.7 pounds). A possible mechanism is cross-selective PPAR-*γ* activation of pterostilbene. This unique response in a small subgroup of patients warrants periodic weight monitoring and further investigation. 

The slight decrease in bicarbonate could indicate a minor acidic effect of pterostilbene in the blood. This is an expected outcome due to the general acidic nature of phenols, such as pterostilbene. This finding does indicate that the encapsulated method of delivery used in this trial appears to be sufficient for blood absorption in humans. 

Some limitations include a small trial population in one region of the United States over approximately 7 weeks. Also, the total daily dose was restricted to 250 mg and no patient reported overuse. While there are no obvious signs of toxicity at this maximum dose, the potential for toxicity cannot be excluded at higher doses.

Neither complete blood count nor urinalysis was performed. Urine was collected for oxidative stress comparison only. Results of a previous short-term healthy volunteer trial demonstrated no baseline changes in blood count when evaluating a pterostilbene-rich extract [8]. The risk of hematological or urinary ADRs was not demonstrated in animal models or a common ADR with currently available PPAR-*α* agonists. There was also no electrocardiogram (ECG) performed due to budgetary constraints. Additional evaluation of ECG monitoring is warranted considering that the target patient population is at-risk for cardiovascular disease and previous dietary supplements have demonstrated QT_c_ prolongation (i.e., Ephedra) [15]. 

In the United States, dietary supplements are not specifically monitored by a regulating body for assessment of quality. Unfortunately, some dietary supplements may contain varying and even absent amounts of listed active ingredients [16]. In this trial, purity was confirmed in a blinded, randomized manner.

## 5. Conclusion

Pterostilbene is generally safe for use in humans at doses up to 250 mg per day. Pterostilbene is well-tolerated at a twice daily dosing frequency. 

## Figures and Tables

**Table 1:** Baseline Demographics.

	High dose	Low dose	Low dose + grape extract	Placebo
Age (years)	54	54	53	54
Gender (%) (M/F)	30/70	25/75	25/75	35/65
Race (%) (CA/AA/Asian)	80/20/0	75/25/0	75/15/10	50/50/0
Smokers (%)	15	15	10	0
Concomitant disease (%)^a^	60	60	40	70
Cholesterol medication use (%)^b^	35	35	35	40
Framingham 10-year risk (%)	6	6	6	6

^a^includes hypertension (overall incidence: 55%), diabetes (5%), atherosclerotic cardiovascular disease (1%), or restrictive/obstructive airway disease (8%).

^b^>75% of the cholesterol medication used in any group were statins.

**Table 2:** Self-reported adverse drug reactions (ADRs)^a^.

	*n*	ADRs reported (%)	*P* value (versus placebo)	Type of ADR (#)
Any pterostilbene	60	10 (16.7)	0.72	—

High-dose pterostilbene	20	5 (25)	0.42	Gastrointestinal (2) Increased appetite (2) Itching (1)
Low-dose pterostilbene	20	3 (15)	1.00	Muscle pain (2) Increased appetite (1)
Low-dose + grape extract	20	2 (10)	1.00	Muscle pain (1) Increased appetite (1)

Placebo	20	2 (10)	—	Gastrointestinal (1) Itching (1)

^a^Intention-to-treat population evaluated.
